# No evidence of amplified *Plasmodium falciparum plasmepsin II* gene copy number in an area with artemisinin-resistant malaria along the China–Myanmar border

**DOI:** 10.1186/s12936-020-03410-6

**Published:** 2020-09-14

**Authors:** Fang Huang, Biraj Shrestha, Hui Liu, Lin-Hua Tang, Shui-Sen Zhou, Xiao-Nong Zhou, Shannon Takala-Harrison, Pascal Ringwald, Myaing M. Nyunt, Christopher V. Plowe

**Affiliations:** 1 National Institute of Parasitic Diseases, Chinese Center for Disease Control and Prevention, Shanghai, People’s Republic of China; 2 Yunnan Institute of Parasitic Diseases, Puer, People’s Republic of China; 3 Center for Vaccine Development and Global Health, University of Maryland School of Medicine, Baltimore, MD USA; 4 Global Malaria Programme, World Health Organization, Geneva, Switzerland; 5 Duke Global Health Institute, Duke University, Durham, NC USA

**Keywords:** *Plasmodium falciparum*, Artemisinin resistance, Piperaquine, *Plasmepsin* II, China–Myanmar border

## Abstract

**Background:**

The emergence and spread of artemisinin resistance in *Plasmodium falciparum* poses a threat to malaria eradication, including China’s plan to eliminate malaria by 2020. Piperaquine (PPQ) resistance has emerged in Cambodia, compromising an important partner drug that is widely used in China in the form of dihydroartemisinin (DHA)-PPQ. Several mutations in a *P. falciparum* gene encoding a kelch protein on chromosome 13 (*k13*) are associated with artemisinin resistance and have arisen spread in the Great Mekong subregion, including the China–Myanmar border. Multiple copies of the *plasmepsin* *II/III* (*pm2/3*) genes, located on chromosome 14, have been shown to be associated with PPQ resistance.

**Methods:**

The therapeutic efficacy of DHA-PPQ for the treatment of uncomplicated *P. falciparum* was evaluated along the China–Myanmar border from 2010 to 2014. The dry blood spots samples collected in the efficacy study prior DHA-PPQ treatment and from the local hospital by passive detection were used to amplify *k13* and *pm2*. Polymorphisms within *k13* were genotyped by capillary sequencing and *pm2* copy number was quantified by relative-quantitative real-time polymerase chain reaction. Treatment outcome was evaluated with the World Health Organization protocol. A linear regression model was used to estimate the association between the day 3 positive rate and *k13* mutation and the relationship of the *pm2* copy number variants and *k13* mutations.

**Results:**

DHA-PPQ was effective for uncomplicated *P. falciparum* infection in Yunnan Province with cure rates > 95%. Twelve non synonymous mutations in the *k13* domain were observed among the 268 samples with the prevalence of 44.0% and the predominant mutation was F446I with a prevalence of 32.8%. Only one sample was observed with multi-copies of *pm2*, including parasites with and without *k13* mutations. The therapeutic efficacy of DHA-PPQ was > 95% along the China–Myanmar border, consistent with the lack of amplification of *pm2.*

**Conclusion:**

DHA-PPQ for uncomplicated *P. falciparum* infection still showed efficacy in an area with artemisinin-resistant malaria along the China–Myanmar border. There was no evidence to show PPQ resistance by clinical study and molecular markers survey. Continued monitoring of the parasite population using molecular markers will be important to track emergence and spread of resistance in this region.

## Background

Malaria remains one of the global major public health problems. According to the latest world malaria report, there were an estimated 228 million cases and 405,000 deaths from malaria globally in 2018, compared with 416,000 estimated deaths in 2017 and 585,000 in 2010 [1].

Artemisinin-based combination therapy (ACT), which combines a fast-acting, rapidly eliminated artemisinin derivative with another slower-acting partner drug with a longer half-life, have played an indispensable role in reducing global malaria-associated mortality and morbidity [2]. Currently, five artemisinin-based combinations are recommended by the World Health Organization (WHO): artesunate–amodiaquine, artemether–lumefantrine, artesunate–mefloquine, artesunate–sulfadoxine–pyrimethamine, and dihydroartemisinin–piperaquine (DHA-PPQ) [3]. ACT has been integral to the recent success of global malaria control and protecting its efficacy for the treatment of malaria has been a global health priority. However, the emergence and spread of artemisinin resistance in *Plasmodium falciparum* poses a threat to malaria control and eradication goals in the Greater Mekong subregion (GMS), where resistance has emerged independently and spread [4–7]. The WHO has implemented a strategy to eliminate *P. falciparum* from the six countries located in the GMS by 2025 to respond the threat of an untreatable multi-drug resistant parasite [8].

Several mutations in a *P. falciparum* gene encoding a kelch protein on chromosome 13 (*k13*) are associated with artemisinin resistance and have arisen multiple times and spread in the GMS, including the China–Myanmar border [9]. Over 200 nonsynonymous *k13* mutations have been reported to date, of which nine variants (F446I, N458Y, M476I, Y493H, R539T, I543T, P553L, R561H, and C580Y) have been validated, and over 20 *k13* mutations are considered candidates or associated markers [9].

From 2010, PPQ became a partner drug with DHA to treat falciparum malaria recommended by the WHO [10]. DHA-PPQ, as the first-line drug for uncomplicated *P. falciparum* treatment, is widely used in China [11] and high efficacy has been reported [12–15]. Recently, the emergence of DHA-PPQ resistance was observed in Cambodia with high treatment failure rate and recrudescent infections. Resistance then rapidly spread to other countries in Southeast Asia [16–22], and subsequent investigations confirmed the presence of high treatment failure and PPQ resistance in Cambodia and Vietnam [16, 21, 23]. The evidence prompted a treatment policy change in Cambodia and Vietnam from DHA-PPQ to other artemisinin-based combinations in areas where DHA-PPQ was failing. Multiple copies of the *P. falciparum plasmepsin* II (*pm2*) genes (PF3D7_1408000), located on chromosome 14, encoding a protease involved in haemoglobin degradation, have recently been shown to be associated with PPQ resistance in Cambodia [19, 24]. This situation raises concern about resistance to the partner drugs of ACT.

There had been zero indigenous malaria infections in China from 2017 and China has vowed to interrupt local malaria transmission by 2020 [25]. Yunnan Province located in Southern China, bordering Myanmar, Laos and Vietnam is the key focus of the national malaria elimination program. In this study, we investigated the therapeutic efficacy of DHA-PPQ for treatment of uncomplicated *P. falciparum* along the China–Myanmar borders from 2010 to 2014. Sequencing of the propeller domain of the *k13* gene was performed to identify mutations and *pm2* copy number was quantified to determine the copy variants from both parasites with and without *k13* mutations.

## Methods

### Study sites and design

**Fig. 1:**
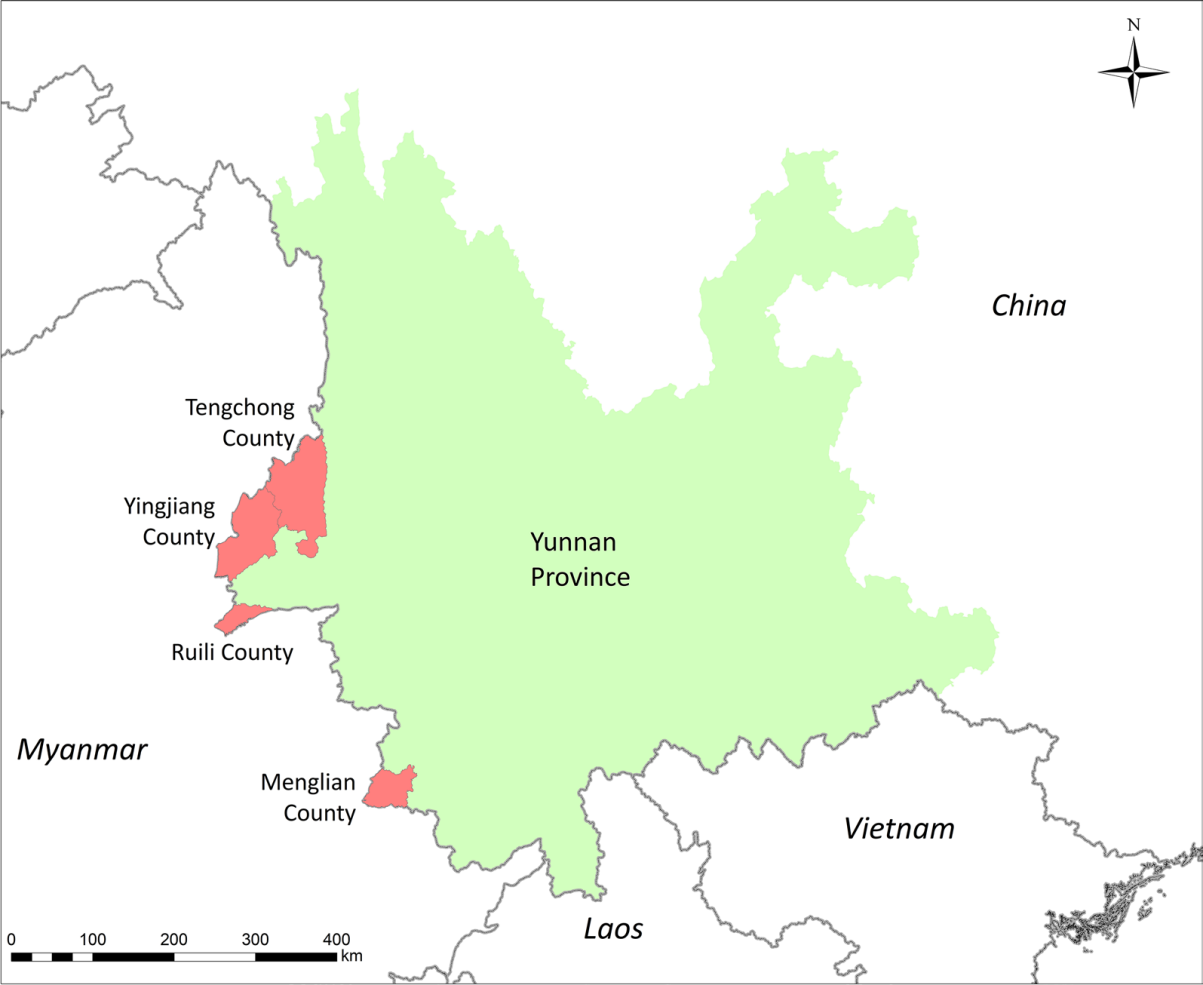
Sentinel sites for therapeutic efficacy study in Yunnan Province from 2010 to 2014

The clinical studies were one-arm prospective evaluations to observe treatment of uncomplicated malaria using a standard WHO therapeutic efficacy study (TES) protocol. The studies were conducted in four counties (Yingjiang, Tengchong, Menglian and Ruili) in Southwest Yunnan along the China–Myanmar border between 2010 and 2014 (Fig. 1).

### Recruitment of patients and follow up

Patients aged > 6 months with fever (axillary temperature **≥ **37.5 ℃) or a history of fever in the previous 48 h were screened for inclusion. Inclusion criteria were: mono-infection with *P. falciparum*; parasitaemia density between 500 and 100,000 asexual parasites/μl; no history of anti-malarial use in the past 14 days and no signs of severe malaria or danger signs. After written informed consent was provided, a detailed medical history, clinical examination and blood smears were performed for each participant. DHA-PPQ (Zhejiang Holley Nanhu Pharmaceutical Co. Ltd, China) was administered at a total adult dose of 2.5 mg/kg dihydroartemisnin and 20 mg/kg PPQ for 3 days. All the anti-malarial drugs were provided by the WHO.

After the first day of treatment (day 0), clinical and laboratory tests, including axillary temperature measurement and thick/thin blood smear preparation and examination were performed at daily for the first 3 days with DHA-PPQ treatment to evaluate the day 3 positive rate. Post-discharge parasite examinations were performed on days 7, 14, 21, 28, 35 and 42. The parasite species and parasitaemia were identified by microscopists certified as Level 1 by the WHO. If the discrepancies of the two values were > 30%, they were re-evaluated by a third microscopist to obtain a final diagnostic consensus (the average of the two close values of the parasitaemia).

### Clinical treatment outcome

Treatment efficacy was evaluated based on clinical and parasitological outcomes and study end points in accordance with WHO guidelines for TES studies (Additional file 1). The outcomes were classified as early treatment failure (ETF), late clinical failure (LCF), late parasitological failure (LPF) and adequate clinical and parasitological response (ACPR). Primary endpoint was ACPR on day 42. PCR genotyping of *msp1*, *msp2* and *glurp*, comparing day 0 and day of failure samples, was used to differentiate recrudescence (same parasite strain) from new infection with another strain parasite [26].

### *k13* sequencing and *pm2* amplification

#### Sample collection and DNA extraction

Dry blood spots on filter paper (Whatman™ 903, GE Healthcare, USA) were collected from participants from TES studies and clinical patients by passive surveillance from 2010 to 2014. All the filter paper samples were used for *k13* sequencing and *pm2* amplification. DNA was extracted from dried blood spots using QIAamp 96 DNA Blood Kit (Valencia, CA, USA).

### *k13* sequencing

A nested PCR amplification method (Takara PCR kit) to amplify the *k13* gene from codon 433 to 702 (850 bp) as described previously with some minor modifications [27]. The primers for nested PCR, sequencing PCR and cycling conditions for *k13* was shown in Additional file 2. PCR products were purified using filter plates (Edge Biosystems, Gaithersburg, MD, USA) and directly sequenced and analyzed on an ABI 3730XL automatic sequencer. The amplification products were analyzed by 1.5% agarose gel electrophoresis before sequencing. Purified products were sequenced by an ABI 3730XL automatic sequencer. Bi-directional sequencing was used and all the products were sequenced twice using independently amplified PCR products.

### *pm2* amplification

ASYBR-green based quantitative PCR (ThermoFisher Scientific, Waltham, MA, USA) was used to determine the copy number of *pm2* (PF3D7_1408000) as previously described with modifications [24]. The copy numbers were quantified with quantitative PCR thermal cycle at 98 °C for 3 min, followed by 45 cycles at 98 °C for 15 s, 63 °C for 20 s, and 72 °C for 20 s on a C1000 Thermal Cycler (Bio-Rad, Marnes-la-Coquette, France) with the CFX96 Real-Time System (Bio-Rad). All the procedures were executed in triplicate. *Plasmodium falciparum β*-*tubulin* (PF3D7_1008700) gene with single copy was used as the internal non-duplicated standard and the *P. falciparum* 3D7 clone as a parallel 1 copy control. The primers sequences for *pm2* and internal standard of *P. falciparum β*-*tubulin* were shown in Table 1. The size of PCR products was 79 bp. The *P. falciparum* NF54 strain was also included in each amplification as the control. The copy number was calculated by the 2-ΔCt method (ΔCt = Ct_*PfPM2*_−Ct_*Pf β*-*tubulin*_, where Ct is the threshold cycle). Copy number > 1.6 was defined as amplification of the gene. The quantitative PCR primers are listed in Table 1 and protocols are shown in Additional file 3.

**Table 1:** *Plasmodium falciparum pm2* copy number determination with listing of quantitative PCR primers and protocol

Primer sequence	Sequences	Tm (℃)	Product size (bp)	Range of melt temperature (℃)
*Pf pm2*_CN_F	5′-TGGTGATGCAGAAGTTGGAG-3′	59.8	79	76.8–77.2
*Pf pm2*_CN _R	5′-TGGGACCCATAAATTAGCAGA-3′	59.4		
*Pf β*-*tubulin*_CN_F	5′-TGATGTGCGCAAGTGATCC-3′	61.9	79	79.0–79.2
*Pf β*-*tubulin*_CN_R	5′-TCCTTTGTGGACATTCTTCCTC-3′	60.5		

### Data analysis

The outcome of the therapeutic efficacy study was evaluated with the WHO protocol [28]. The output sequence data of *k13* were assembled, edited, and aligned using Sequencher (version 5.1) software. PlasmoDB accession number PF3D7_1343700 (http://www.plasmodb.org) was used as a reference sequence. Fisher’s exact test was used for statistical analysis. A regression test was used to estimate the association between the day 3 positive rate and *k13* mutation and the relationship of the *pm2* copy number variants (CNV) and *k13* mutations. Variables with *P* < 0.05 were considered statistically significant.

### Ethical considerations

This study was approved by the Institutional Review Board of the National Institute of Parasitic Diseases, Chinese Center for Disease Control and Prevention and of the WHO Western Pacific Regional Office. Written informed consent was obtained from patients or guardians. The studies were also registered as clinical trials at https://www.anzctr.org.au under the numbers ACTRN12610001008011 and ACTRN12610001028099.

## Results

### Therapeutic efficacy study

A total of 174 participants received directly observed anti-malarial treatment with DHA-PPQ. The treatment outcome of DHA-PPQ was adjusted and corrected by PCR [29]. One hundred and forty of 174 participants completed the 42 days follow-up and 19.5% (34/174) were withdrawn or lost to follow-up. The ACPR rates in Yingjiang of 2010 (20/20), 2012(43/43) and 2013(20/20), Tengchong in 2012 (20/20) and Ruili in 2013 (6/6) were all 100.0%. The average ACPR rate of DHA-PPQ treatment in all the sites was 99.5% (minimum: 96.8%; maximum: 100.0%) (Table 2). One patient from Menglian County in 2014 showed treatment failure after DHA-PPQ treatment on day 35 was confirmed using PCR amplification as *P. vivax* infection. Another participant from Yingjiang County in 2012 experienced early treatment failure with parasitaemia and axillary temperature ≥ 37.5 °C on day 3 following treatment with DHA-PPQ, but this patient cleared parasitaemia without rescue treatment and was finally classified to be ACPR [26]. Seven of 174 participants (4.0%) were positive for parasitaemia on day 3 after DHA-PPQ treatment.

**Table 2:** Outcome of the therapeutic efficacy study of DHA-PPQ treatment of uncomplicated *P. falciparum* from 2010 to 2014

Year	Sites	Day 3 + (%)	ACPR (%)	LCF (%)	LPF (%)	ETF (%)	WTH/LFU (%)
2010^c^	Yingjiang	5.9% (1/29)	100.0% (20/20)	0.0% (0/20)	0.0% (0/20)	0.0% (0/20)	31.0% (9/29)
2012^c^	Yingjiang	3.9% (2/50)	100.0% (43/43)^a^	0.0% (0/43)	0.0% (0/43)	0.0% (0/43)	14.0% (7/50)
2012^c^	Tengchong	4.5% (1/22)	100.0% (20/20)	0.0% (0/20)	0.0% (0/20)	0.0% (0/20)	9.1% (2/22)
2013	Yingjiang	9.5% (2/22)	100.0% (20/20)	0.0% (0/20)	0.0% (0/20)	0.0% (0/20)	9.1% (2/22)
2013	Ruili	0.0% (0/11)	100.0% (6/6)	0.0% (0/6)	0.0% (0/6)	0.0% (0/6)	45.5% (5/11)
2014	Menglian	2.5% (1/40)	96.8% (30/31)	3.1% (1/31)^b^	0.0% (0/31)	0.0% (0/31)	22.5% (9/40)

ACPR, adequate clinical parasitological response; LCF, late clinical failure; LPF, late parasitological failure; ETF, early treatment failure; WTH, withdrawal; LFU, loss to follow-up

^a^One of 43 participants had parasite and fever on day 3, but this case was continually followed up and cleared parasitaemia without rescue treatment and was finally classified to be ACPR

^b^*P. vivax* infection was positive on day 35

^**c**^Part data of the therapeutic efficacy study of DHA-PPQ has been presented in another study [7]

### *k13* sequencing

The *k13* gene was successfully sequenced in 268 samples from therapeutic efficacy studies (39.6%, 99/268) and the hospitals by passive surveillance (63.1%, 169/268) (Table 3). Twelve non synonymous mutations (F446I, N458Y, Q467H, C469Y, F483S, F495L, P553L, E556D, R561H, A578S, V589G, and Q661R) in the *k13* propeller domain were observed in 118 samples, and the prevalence of *k13* mutations was 44.0% (118/268). The prevalence of *k13* mutation in TES samples and passive surveillance was 46.5% (46/99) and 46.2% (72/169), respectively. F446I was the predominant *k13* mutation and accounted for 74.6% (88/118) of all *k13* mutations. The *k13* mutation from samples collected from Yingjiang and Tengchong had a higher prevalent than from Ruili and Menglian. In addition, five of the seven patients who were positive for parasitaemia on day 3 after DHA-PPQ treatment were observed with *k13* mutation (N458Y, C469Y, and P553L). The *k13* mutation was not significantly associated with the day 3 positive parasitaemia after DHA-PPQ treatment (*P *= 0.245).

**Table 3:** Information of sample collection and prevalence of *k13* mutation and *pm2* with multi-copies in all the samples

Sample collection	Year	Site	*k13*			*pm2*					
			No. of tested	No. of mutant	% of mutant	No. of tested	No. of *k13* wild-type	No. of *k13* mutant	Single copy	Multi-copies (> 1.6)	% Of multi-copies
TES	2010	Yingjiang	26	16	61.5%	20	10	10	20	0	0.0%
	2012	Tengchong	12	10	83.3%	10	1	9	10	0	0.0%
	2012	Yingjiang	25	9	36.0%	20	16	4	20	0	0.0%
	2013	Yingjiang	20	11	55.0%	19	9	10	19	0	0.0%
	2013	Ruili	6	0	0.0%	6	6	0	6	0	0.0%
	2014	Menglian	10	0	0.0%	10	10	0	10	0	0.0%
		Sub total	99	46	46.5%	85	52	33	85	0	0.0%
Passive detection	2011	Menglian County hospital	8	0	0.0%	–	–	–	–	–	
	2011	Tengchong (Wuhe hospital)	125	49	39.0%	105	68	37	105	0	0.0%
	2012	Yingjiang (Nabang Hospital)	4	2	50.0%	3	2	1	3	0	0.0%
	2013	Tengchong County hospital	6	4	66. 7%	6	2	4	6	0	0.0%
	2013	Yingjiang (Nabang Hospital)	12	11	91.7%	12	1	11	11	1^a^	9.1%
	2014	Yingjiang (Nabang Hospital)	14	6	42.9%	13	8	5	13	0	0.0%
		Sub total	169	72	42.6%	139	81	58	138	11	9.1%
Total			268	118	44.0%	224	133	91	223	1	0.4%

^a^The only one sample with multi-copies of *pm2* was carrying *k13* mutant

### *pm2* copy number variation

**Fig. 2:**
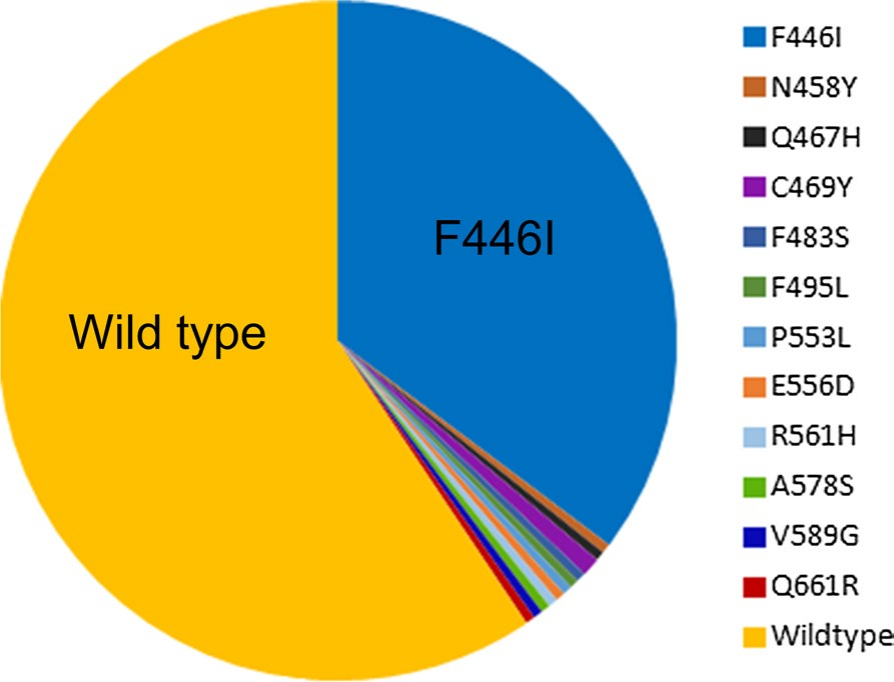
Proportion of *k13* polymorphisms of the samples tested on *pm2* amplification

The 224 samples comprising 91 with *k13* mutant and 133 with *k13* wild-type were tested on *pm2* copy number amplification (Table 3). Proportion of different *k13* genotype of the samples for *pm2* amplification was shown in Fig. 2. Only one sample was observed with multi-copies of *pm2* from *k13* mutant samples and other *k13* mutant or wild-type samples showed no copy variants of *pm2* (99.6%, 223/224). These data were consistent with the therapeutic efficacy of DHA-PPQ. Since there was only one sample with *pm2* multiple copies, the data did not meet the conditions for statistical analysis of the association between the *pm2* copy number variants and *k13* mutations. Nevertheless, it was necessary to note that this sample with multi-copies of *pm2* was also carrying *k13* mutation.

## Discussion

The emergence and rapid spread of multidrug-resistant *P. falciparum* across the GMS is a threat to the therapeutic efficacy of DHA-PPQ for treatment of uncomplicated *P. falciparum*. According to the WHO definition, the partial resistance to artemisinin is characterized as much slower clearance of parasitaemia in the first 3 days of treatment following artemisinin mono-therapy or ACT [30]. Such resistance does not usually lead to treatment failure; however, if the artemisinin component is less effective, the partner drug has to clear a greater parasitic mass, jeopardizing the future efficacy of the partner drug. PPQ resistance was first reported in Cambodia [20]. Recently, alarmingly high rates of treatment failure have occurred in Cambodia, Vietnam, and Thailand, which will have contributed to increased transmission of *P. falciparum* [21].

In Africa, artemisinin resistance emergence has been reported [31], but artemisinin (partial) resistance has not been confirmed to date and first-line ACT remains efficacious in all malaria-endemic settings [32–34]. DHA-PPQ was increasingly deployed as an anti-malarial drugs in Africa. The *pm2* duplications was observed in recurrent infections in Mali within 2 months after DHA-PPQ treatment [35]. Another study reported that only a single copy of *pm2* was detected in two isolates from Ethiopia and Cameroon after DHA-PPQ failures [36, 37]. These findings indicated the DHA-PPQ failures or *pm2* multi-copies in very few cases in Africa, but still raised concerns about the long-term efficacy of DHA-PPQ treatment.

In a previous study, *P. falciparum* showed markedly delayed clearance following 7 days’ treatment with artesunate along the China–Myanmar border, and *k13* F446I was the predominant mutation and was associated with delayed parasite clearance [7]. The present study aimed to evaluate the therapeutic efficacy of DHA-PPQ, which is the most widely used artemisinin-based combinations in China, as the first-line drug for uncomplicated *P. falciparum* infection. DHA-PPQ was still efficacy in Southern Yunnan Province with > 95% ACPR outcome. In the 1970s to 1980s, PPQ alone was used for prophylaxis and treatment in the malaria endemic areas in Southern China [15, 38]. In 2009, PPQ was used as a partner drug of artemisinin for first-line treatment of *P. falciparum* infection in China and now DHA-PPQ is the most widely used artemisinin-based combination. Although no resistance to artemisinin and its partner drug PPQ was confirmed in falciparum malaria in Yunnan Province, the efficacy of ACT was decreasing. The prevalence of day 3 positive parasitaemia was > 3% in most of the sentinel sites and artemisinin resistance has been confirmed along the China–Myanmar border [7, 13].

Only one sample was observed with *pm2* multi-copies with *k13* mutation out of 91 samples and no copy variants of *k13* wild-type samples. The association between *k13* polymorphisms and *pm2* amplification was strong in the Cambodian parasites, which reflected the history of drug selection in Cambodia [24]. The isolates from the China–Myanmar border in our study showed high prevalence of single copy of *pm2* compared with the high multi-copies in Cambodia, which also indicated the low connectivity of genetic population between parasites from the China–Myanmar border and Cambodia [39]. Furthermore, the data for *pm2* copy number variants provide evidence that no PPQ resistance has emerged along the China–Myanmar border, which is consistent with the therapeutic efficacy of DHA-PPQ.

China is approaching malaria elimination and zero indigenous cases were reported from 2017 [25, 40]. There are still several challenges in the post malaria elimination stage. One big challenge is how to maintain surveillance and response capacity. Monitoring the efficacy of anti-malarial drugs for the treatment of imported cases was recommended by the WHO in “A framework for malaria elimination” [41], which was called integrating drug efficacy surveillance (iDES). China has set up an anti-malarial drug surveillance network that is responsible for implementing iDES of DHA-PPQ and anti-malarial molecular markers surveillance throughout the country.

## Limitations

This study only analyzed the *pm2* copy number variants of *P. falciparum* isolates along the China–Myanmar border. Recent studies have identified that mutations in *P. falciparum* chloroquine resistance transporter (*pfcrt*) could contribute to a multi-factorial basis of PPQ resistance [42–44], which indicates that there are multi-genes or multi-mutations involved in PPQ resistance. Therefore, further molecular surveillance of PPQ resistance including multi-genes will be necessary.

## Conclusion

DHA-PPQ for uncomplicated *P. falciparum* infection was still efficacy in the area with artemisinin-resistant malaria along the China–Myanmar border. There was no evidence to show PPQ resistance in the clinical study and molecular markers survey of *pm2*. Continued monitoring of the parasite population using molecular markers will be important to track emergence and spread of resistance in this geographic region.

## Supplementary information

**Additional file 1. **Classification of treatment outcomes in the therapeutic study.

**Additional file 2: Table S1.** Primers for nested PCR and sequencing PCR and cycling conditions for *k13*.

**Additional file 3. **The protocol for *pm2* copy number amplification.
