# Supplementary material for: No evidence of amplified Plasmodium falciparum plasmepsin II gene copy number in an area with artemisinin-resistant malaria along the China–Myanmar border
Source: Malar J. 2020 Sep 14;19:334. doi: 10.1186/s12936-020-03410-6 (PMC7488220; doi:10.1186/s12936-020-03410-6)
Supplement: Supplementary file 1 — Additional file 1. Classification of treatment outcomes in the therapeutic study. [file 12936_2020_3410_MOESM1_ESM.docx]

**Supplementary File 3**

**Classification of treatment outcomes**

***Early treatment failure (ETR)***

- Danger signs or severe malaria on day 1, 2 or 3 in the presence of parasitaemia;
- Parasitaemia on day 2 higher than on day 0, irrespective of axillary temperature;
- Parasitaemia on day 3 with axillary temperature ≥ 37.5 ºC;
- Parasitaemia on day 3 ≥ 25% of count on day 0.

***Late clinical failure (LCF)***

- Danger signs or severe malaria in the presence of parasitaemia on any day between day 4 and day 28 (day 42) in patients who did not previously meet any of the criteria of early treatment failure;
- Presence of parasitaemia on any day between day 4 and day 28 with axillary temperature ≥ 37.5 ºC (or history of fever) in patients who did not previously meet any of the criteria of early treatment failure

***Late parasitological failure (LPF)***

- Presence of parasitaemia on any day between day 7 and day 28 with axillary temperature < 37.5 ºC in patients who did not previously meet any of the criteria of early treatment failure or late clinical failure

***Adequate clinical and parasitological response (ACPR)***

- Absence of parasitaemia on day 28, irrespective of axillary temperature, in patients who did not previously meet any of the criteria of early treatment failure, late clinical failure or late parasitological failure
