# Supplementary material for: No evidence of amplified Plasmodium falciparum plasmepsin II gene copy number in an area with artemisinin-resistant malaria along the China–Myanmar border
Source: Malar J. 2020 Sep 14;19:334. doi: 10.1186/s12936-020-03410-6 (PMC7488220; doi:10.1186/s12936-020-03410-6)
Supplement: Supplementary file 2 — Additional file 2: Table S1. Primers for nested PCR and sequencing PCR and cycling conditions for k13. [file 12936_2020_3410_MOESM2_ESM.docx]

Table S1 Primers for nested PCR and sequencing PCR and cycling conditions for K13

| Primer sequences | Amplification conditions | Size of PCR products |
| --- | --- | --- |
| K1-F: 5’- cggagtgaccaaatctggga-3’  K4-R: 5’- gggaatctggtggtaacagc-3’ | 95^o^C×2 min, 30 cycles of 95^o^C×30 sec, 60^o^C×90 sec, 72^o^C × 90 sec], 72^o^C ×10 min | 2097bp |
| K2-F: 5’- gccaagctgccattcatttg -3’  K3-R: 5’-gccttgttgaaagaagcaga-3’ | 95^o^C×2 min, 30 cycles of 95^o^C×30 sec, 60^o^C×90 sec, 72^o^C × 90 sec], 72^o^C ×10 min | 850bp |
| K2-F:5’- gccaagctgccattcatttg -3’  K3-R:5’-gccttgttgaaagaagcaga-3’  K5-F:5’-ttatgtcattggtggaactaa-3’  K6-R:5’-tctaggggtattcaaaggtgc-3’ | Purified products were sequenced by an ABI 3730XL automatic sequencer |  |
